# Soil environmental quality in Nanling commodity grain base based on equal intercept transformation radar chart

**DOI:** 10.1038/s41598-021-90103-y

**Published:** 2021-05-17

**Authors:** Hai-biao Dong, Guang-hui Zhang, Ming-jiang Yan, Yan-liang Tian

**Affiliations:** 1 The Institute of Hydrogeology and Environmental Geology, Chinese Academy of Geological Sciences, Shijiazhuang, 050000 China; 2 Key Laboratory of Groundwater Sciences and Engineering, Ministry of Natural Resources, Shijiazhuang, 050000 China

**Keywords:** Environmental sciences, Environmental chemistry, Environmental impact

## Abstract

This paper introduces for the first time the equal intercept transformation radar chart—an improved form—to the assessment of soil environmental quality of Nanling commodity grain base. The equal intercept transformation radar chart, a visual graphical data analysis method, translates data from a numerical to graphical format. This visualization enables data presentation, analysis process and results stick out a mile and is capable of fully retaining information contained in data and excavating it in depth from geometry. Moreover, it overcomes pertinently the main defect of the conventional radar chart that the evaluation result depends heavily on the order of arrangement of indicators. The results indicated that the soil environmental quality at depths of 0–60 cm in the low mountain area of the Nanling commodity grain base was the second grade, while that in the hilly and plain areas were both first grade. The indicators of poor soil environmental quality in the low mountain area were exogenous Cd and endogenous As; those in the hilly area were exogenous Cd and endogenous As and Hg; and that in the plain area was exogenous Cd. The results were in line with the actual situation of the study area.

## Introduction

Nanling important commodity grain base is an important component of the Wan Jiang Economic Belt and is a national high-standard farmland demonstration area that produces high-quality rice and vegetables. The selection of planting patterns and the consistent production of high crop yields are largely controlled by the soil properties and quality of the base. Soil quality can be defined as the capacity of a specific type of soil to sustain plant and animal productivity, maintain environmental quality and support human health and habitation within natural or managed boundaries^1^. Soil quality integrates inherent and dynamic soil properties and is influenced by land use and management practices that interacts with the soil system^2^. Soil environmental quality, as an important component of soil quality, reflects the level of harmful substances in the soil. The scientific, accurate and comprehensive assessment of the soil environmental quality of the study area has important significance for planning land resources rationally, developing high-standard farmland, enhancing characteristic agriculture and improving the quality and efficiency of agricultural production^3,4^.

Many methods and various models have been used for soil quality assessment^5–8^. The commonly used methods of soil environment quality assessment include the single factor index method, comprehensive index method, fuzzy mathematics method, multivariate statistical analysis and artificial neural network method.

The comprehensive index method has been successfully used to assess soil quality in many regions, at different scales and under different agricultural management practices^6,9–12^. Weissmannová and Pavlovský^13^ provided a review of assessments of soil quality using various indices. The indices were divided into individual indices, and total comprehensive indices and the calculation formulas for every index, along with the classes of contamination or risk of soil indicated by the corresponding index value, were presented. This method can only provide the grade of soil quality and is unable to display the difference in indicator content, thereby losing some unique information originally present in the data.

Hu et al*.*^14^ applied the fuzzy mathematical method to the environmental risk assessment of soil at a petroleum-contaminated site in China and distinguished the primary environmental risk in the soil. This method considers the fuzziness of evaluation, but the determination of fuzzy weight is subjective, which directly affects the reliability of evaluation results. Singh et al*.*^15^ performed an environmental risk assessment of heavy metal pollution using multivariate analysis in the soils of Varanasi, India, and identified the principal contaminants. However, this method has shortcomings in terms of classification and consistency checks. Liu et al*.*^16^ assessed the soil quality of soil polluted with heavy metals in Tai Yuan city based on the support vector machine method and provided a classification of the soil quality. This type of intelligent algorithm has a strong ability to build mapping relationships, but the model training and computation processes are complex.

A radar chart, which refers to charts that resemble navigation radar graphics and are also known as spider charts, is a graphical method of data analysis. In this method, the value of multiple related attributes are drawn by a certain method; then, through analysis of drawn charts, the subject of analysis can be comprehensively evaluated. Radar chart are mainly used in the evaluation of an enterprise's financial condition, operation risk assessment and so on.

Du^17^ applied the radar chart method to audit the economic benefits of two companies, obtained evaluation results, and analyzed the reasons to provide corresponding suggestions.

In the fields of the Earth sciences and environmental quality, the radar chart method has rarely been used: Zhang et al*.*^18^ applied a radar chart to distinguish basalt tectonic environments and trace mineral source areas and analyzed its applicability and ability to achieve certain effects. Zhang^19^ compared and analyzed the environmental quality of different regions and different years using the radar chart method and obtained a comprehensive environmental index. In the assessment of soil environmental quality, the use of radar charts is still rare. Besides, there is a major weakness in traditional radar chart method that the evaluation result varies wildly from one order of arrangement of indicators to another.

The equal intercept transformation radar chart could effectively address this disadvantage by adding equal intercept axes. There are very few works published on the equal intercept transformation radar chart^20^, not to mention application of it to study of soil environmental quality.

In order to verify the applicability of equal intercept transformation radar chart and improve the evaluation results, this paper introduced originally the equal intercept transformation radar charts in the assessment of the environmental quality of shallow soil in different landforms and depths in the Nanling commodity grain base. In the assessment, the equal intercept transformation radar chart area was used to represent the soil environmental quality level.

Unlike other assessment methods of soil environment quality in common use, the equal intercept transformation radar chart, as a visual graphical data analysis method, translates data from a numerical to graphical format. This visualization enables data presentation, analysis process and results intuitive and is capable of fully retaining information contained data and examining it in depth from geometry. Meanwhile, the equal intercept transformation radar chart is totally independent of the orders of arrangement of indicators^20–23^.

This study could broaden the scope of application of equal intercept transformation radar chart and improve the evaluation of soil environmental quality further.

## Results

**Figure 1:**
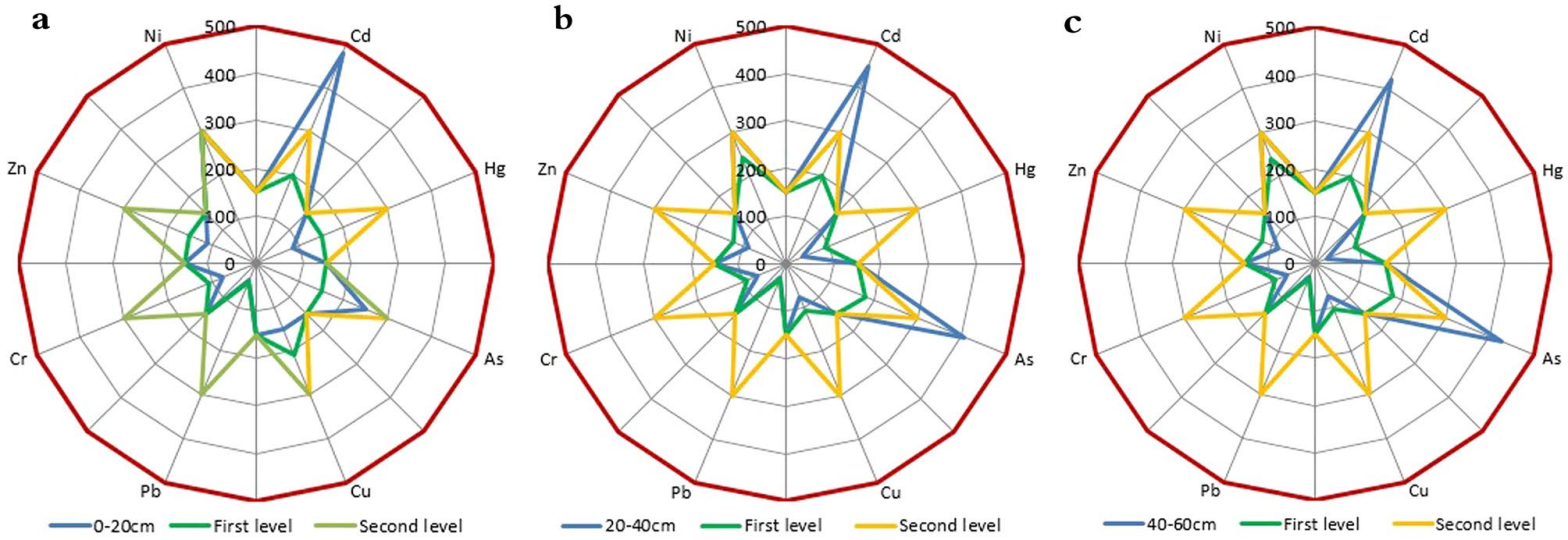
Environmental quality equal intercept transformation radar chart of the soil in the low mountainous area.

**Figure 2:**
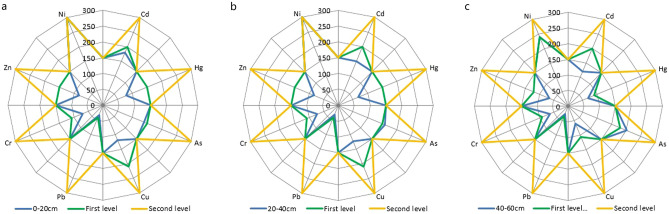
Environmental quality equal intercept transformation radar chart of the soil in the hilly area.

**Figure 3:**
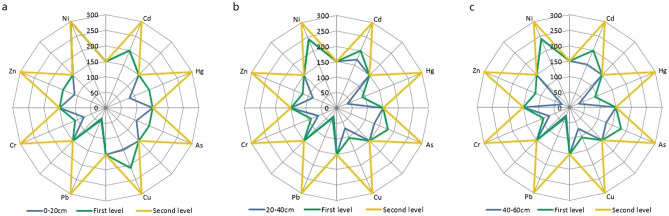
Environmental quality equal intercept transformation radar chart of the soil in the plain area.

The equal intercept transformation radar charts of soil environmental quality at different depths in the low mountain area, hilly area and plain area of the study area are shown Figs. 1, 2 and 3.

### Area analysis of the equal intercept transformation radar charts

The area of an equal intercept transformation radar chart represents the soil environmental quality. The areas of the above equal intercept transformation radar charts are shown in Table 1.

**Table 1:** Areas of the equal intercept transformation radar charts.

	Low mountain	Hilly area	Plain area
0–20 cm	153,486.10	125,736.07	128,539.04
20–40 cm	151,161.69	124,648.11	115,603.52
40–60 cm	149,724.75	116,579.18	111,043.29
First level grade	128,820		
Second level grade	205,200		

The areas of the soil environmental quality equal intercept transformation radar charts of various geomorphic units at different depths were compared with the standard radar chart. If the measured area is less than the standard radar chart area of the first grade, the soil environmental quality level of the corresponding geomorphic unit and corresponding depth is classified as the first grade. If the area is between the standard radar chart area of the first grade and that of the second grade, the soil environmental quality level of the corresponding geomorphic unit and corresponding depth is classified as the second grade. According to this, the soil environmental quality level in each depth on each geomorphic unit can be obtained (see Table 2).

**Table 2:** Soil environmental quality level of the each depth of the each geomorphic unit.

	Low mountain	Hilly area	Plain area
0–20 cm	Second level	First level	First level
20–40 cm	Second level	First level	First level
40–60 cm	Second level	First level	First level

As shown in Table 2, the soil environmental quality of 0–60 cm in the low mountain area is second grade, which can be described as “still clean” and generally pollution-free. The soil environmental quality of 0–60 cm in both the hilly area and plain area is the first grade, which is clean and pollution-free.

**Figure 4:**
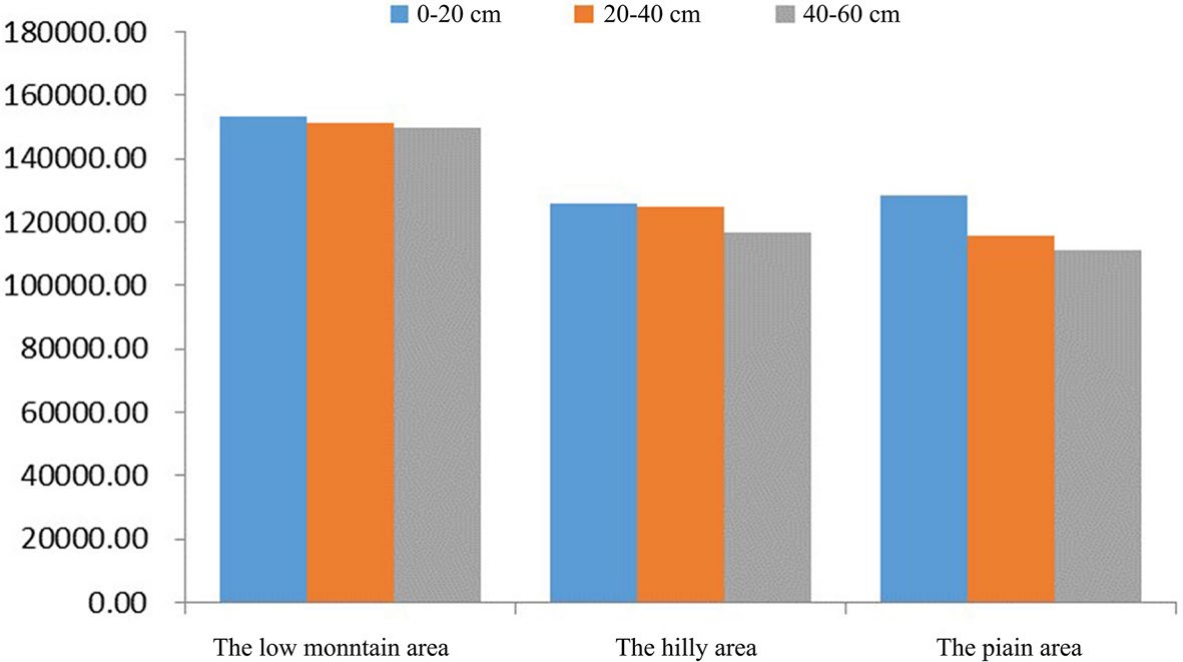
The soil environmental quality (represented by areas) of each depth of each geomorphic unit.

The areas of the soil environmental quality equal intercept transformation radar charts of different geomorphic units and different depths were drawn on a columnar map (shown Fig. 4).

As seen from Fig. 4, in space, there is not much difference in soil environmental quality between the hilly area and the plain area, which are both superior in environmental quality to the low mountain area. Specifically, the soil environmental quality of 0–20 cm is ranked as hilly area > plain area > low mountain area and that of 20–60 cm is ranked as plain area > hilly area > low mountain area.

In the vertical direction, with increasing depth, the soil environmental quality in the low mountain area, hilly area and plain area gradually increased. In the hilly area and plain area, the soil environment quality changed greatly, while that in the hilly area changed little. The soil environmental quality in the hilly area increased significantly from 20–40 cm to 40–60 cm, while the increase in the plain area occurred from 0–20 cm to 20–40 cm.

### Analysis of each factor

The soil environmental quality of 0–20 cm is ranked as hilly area > plain area > low mountain area and that of 20–60 cm is ranked as plain area > hilly area > low mountain area.

As shown in Figs. 1, 2 and 3, the indicators of poor soil environmental quality of shallow soil in the low mountain area are Cd and As, those in the hilly area are Cd, As and Hg and that in the plain area is Cd.

At the depth of 0–20 cm, the mining of gold and copper mines with high Cd in low mountain areas and the application of phosphate fertilizers with high Cd content have significantly increased the Cd content in the soil in the low mountain area. In addition, the soil in the low mountain area is mostly lime soil, of which the parent material is carbonate weathering, with a high As content. Therefore, the soil environmental quality in the low mountain area is the worst.

The plain area is flat and open with developed water system and deep soil layers, where a large area of farmland is distributed. The long-term and large-scale application of phosphorus fertilizer with high Cd content makes the Cd content in the farmland soil in the plain area higher and the soil environment quality worse.

In comparison, the imported Cd in the soil in the hilly area is lower and the soil environmental quality is the best.

Below 20 cm depth, with increasing depth, the soil particle size gradually decreases, and the clay mineral content in the soil gradually increases. The elements content mainly derived from the parent material of the soil gradually increase. For example, the endogenous Cd in the low mountain soil increases with depth. The parent material of soil in hilly area is mostly weathered sand conglomerate, in which the content of As and Hg is high. With the increase of depth, the contents of As and Hg in the hilly area soil increase. On the other hand, the exogenous elements are mainly adsorbed on the surface of clay minerals and soil organic matter. With the increase of depth, although the clay mineral content increases to some extent, the organic matter content decreases significantly, so the imported Cd content in the plain area soil decreases. Therefore, the soil environmental quality in the low mountain area is still the worst, while that in the plain area surpasses the hilly area.

Beyond the above elements, the contents of Cu, Pb, Cr, Zn and Ni are generally low on the whole, which are stable or in a good trend vertically.

After analysis, the results are in line with the actual situation of the site.

### Comparison with other common evaluation methods

The commonly used single factor index method, comprehensive index method, fuzzy mathematics method, multivariate statistical analysis and artificial neural network method, etc. are analytical data analysis methods. Utilizing some analytic means, only numerical outcomes can be obtained based on the data, which is non-graphic and thus abstract and invisible and probably obscures uniqueness of data.

In sharp contrast, the equal intercept transformation radar chart is a visual graphical data analysis method and translates data from a numerical to graphical format. This visualization enables data presentation, analysis process and results stick out a mile and is capable of fully retaining information contained in data and excavating it in depth from geometry.

**Figure 5:**
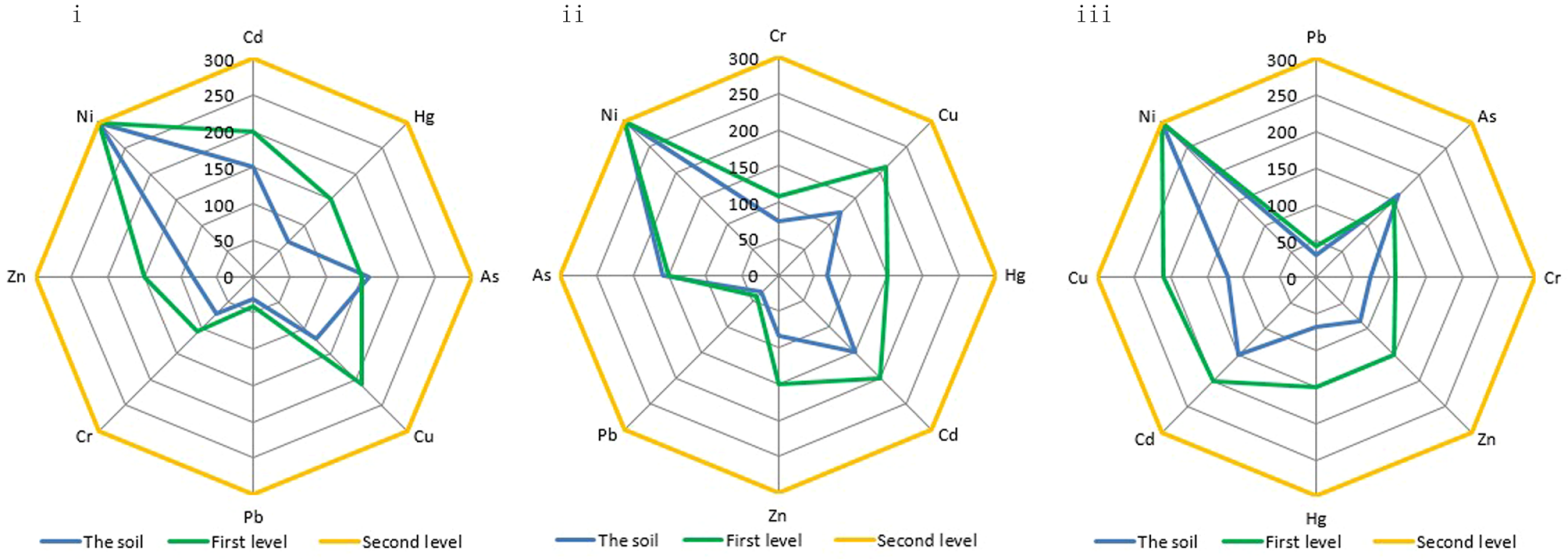
Environmental quality radar charts of 20–40 cm soil in the hilly area with different indicator orders.

Furthermore, there is a main defect in conventional radar chart that the evaluation depends heavily on the orders of arrangement of indicators. Taking 20–40 cm soil in the hilly area as an example, Fig. 5 shows any three orders of arrangement of indicators of all. The shapes of the radar charts are quite different and there are huge gaps among their areas.

The equal intercept transformation radar chart-an advanced form-settles it pertinently. Each set of data has only one corresponding equal intercept transformation radar chart, greatly facilitating the comparisons among various samples and relevant standards.

## Discussion

Two major conclusions regarding the study of soil environmental quality can be drawn from our observations using equal intercept transformation radar chart method. First, the soil environmental quality at depths of 0–60 cm in the low mountain area of Nanling commodity grain base is second grade, while that in the hilly and plain areas is first grade. The soil environmental quality of 0–20 cm is ranked as hilly area > plain area > low mountain area and that of 20–60 cm is ranked as plain area > hilly area > low mountain area. Second, the indicators of poor soil environmental quality of shallow soil in the low mountain area are imported Cd from mining of gold and copper mines with high Cd in there and the application of phosphate fertilizers with high Cd content and endogenous As from parent material of the soil-carbonate weathering, those in the hilly area are exogenous Cd and endogenous As and Hg from parent material of the soil-weathered sand conglomerate and that in the plain area is exogenous Cd from long-term and large-scale application of phosphorus fertilizer with high Cd content. These are in line with the actual conditions of the site.

This paper originally applied the equal intercept transformation radar chart method to the assessment of soil environmental quality. The special case study presented here shows that the method is feasible and that the method is feasible has unique advantages. The equal intercept transformation radar chart method translates data from a numerical to graphical format, which enables data presentation, analysis process and results stick out a mile and is capable of fully retaining information contained data and excavating it in depth from geometry. Moreover, it overcomes pertinently the main defect of the conventional radar chart that the evaluation result depends heavily on the order of arrangement of indicators and thus largely facilitating the comparisons among various samples and relevant standards.

In future research, we could analyze other geometric attributes of the equal intercept transformation radar chart and identify their meanings to further extract information and further enrich the assessment. In addition, intercept selection, data scaling, correlation between indicators and arc charts would be the potentials.

## Methods

### Site

**Figure 6:**
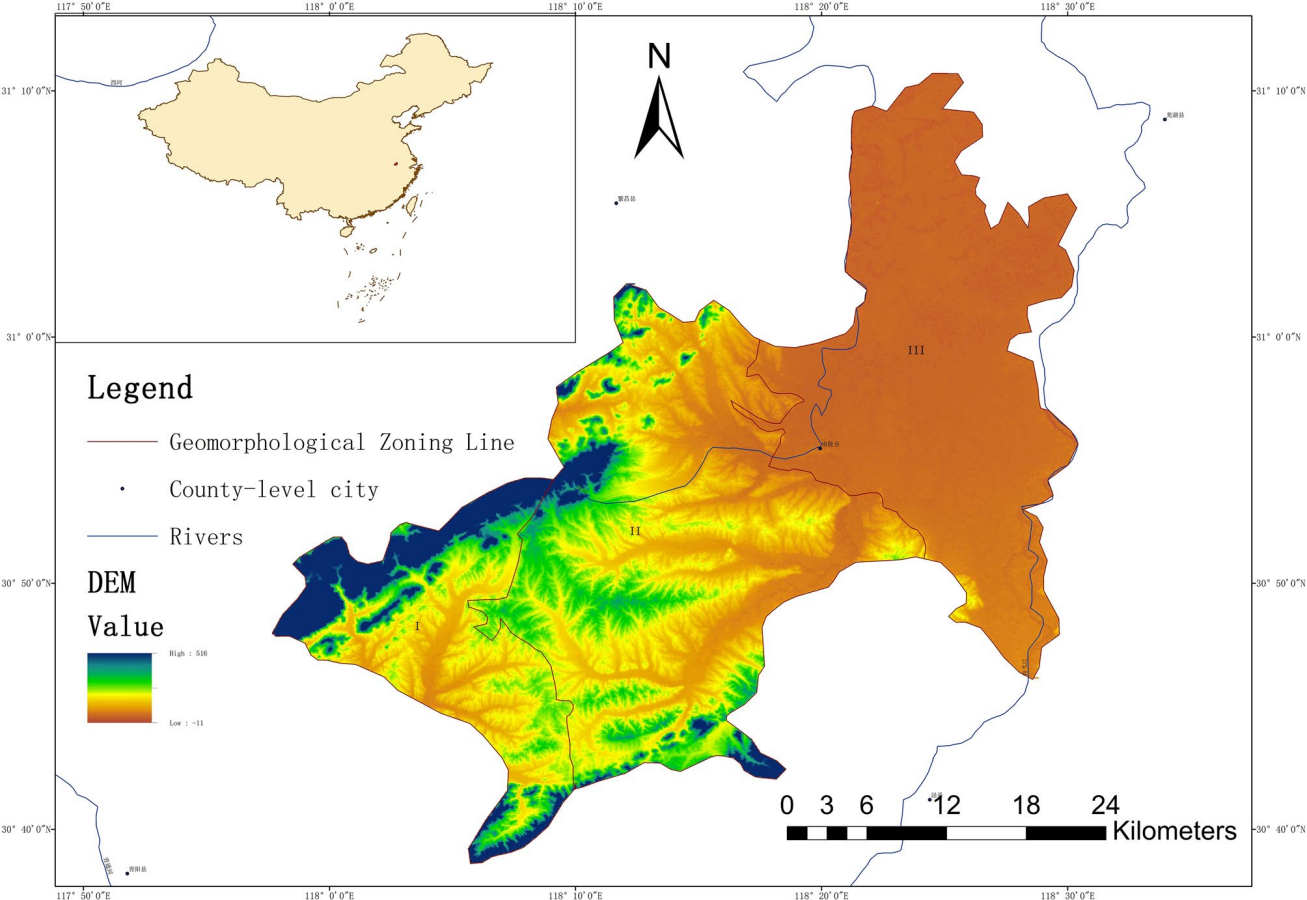
Topographic and geomorphological maps of Nanling area (Mapgis 6.7 http://10.90.90.90/#/portal/home).

Nanling national important commodity grain base is located in southeastern of Anhui Province and has geographic coordinates of 117°30′57″–118°31′30″E, 30°35′20″–31°10′40″N and a total area of approximately 880 km^2^ (see Fig. 6).

The site is located in the humid monsoon climate zone of the northern subtropical zone and features high temperatures and abundant rainfall in June and July. The research area lies within the Yangtze River basin, and the rivers flowing through the area are mainly the Qingyi River and Zhang River and there many tributaries. As the largest tributary of the middle and lower reaches of the Yangtze River, the Qingyi River flows from the south to the east of the study area, and it is the base level towards which surface water and groundwater drain in the study area. The Zhang River originates in the site, and the general flow is from south to north. The two rivers are connected by the Zifu River. In the northeast of the study area, the rivers and lakes are connected, and a water network is developed.

The site is located in the transitional zone between the mountainous area of southern Anhui Province and the plains along the Yangtze River. In general, the topography is the highest in the southwest and gradually decreases to the northeast, forming low mountain areas (I), hilly areas (II) and plain areas (III). The soil types in the study area are mainly acidic paddy soils.

The site is an important part of the Wan Jiang Economic Zone. Nanling County is the main grain-producing area in Anhui Province, and rice and tea are abundant in this site. Scientific evaluation of soil environmental quality is therefore critical to high-standard farmland planning and development in this site.

### The equal intercept transformation radar chart

The equal intercept transformation radar chart is an improved form of conventional radar chart by adding equal intercept axes^20^. Its basic elements include: first, N rays drew with the same angle from the same origin as figure axis respectively represent the N indicators of the evaluation objects; second, a fixed length line segment was cut on the bisector of every two adjacent index axes, i.e. “equal intercept”; third, With the coordinates of each index value, the N points are made on the corresponding axis; last, the N points and N ends of intercepts are connected successively by line segments to form a 2N-side polygon, equal intercept transformation radar chart.

It is suitable for the comprehensive evaluation of an object composed of several indicators. In the application, the distances from the origin to the end of different axes should not vary too much. If the gaps between different indicators are too large, it needs to be scaled by a certain method so that the corresponding length of the line segments on the graph is in the same order of magnitude. The values represented by the coordinate ranges and unit lengths of different axial directions may be different to suit the actual values of different indicators.

### Assessment processes

This paper used 1962 groups of soil samples collected from a 1:5 million land quality geochemical survey and selected the eight indicators of Cd, Hg, As, Cu, Pb, Cr, Zn and Ni according to the People's Republic of China soil environmental quality standard (GB15618-1995 and GB15618-2008 referred) to explore the application of equal intercept transformation radar charts in the assessment of the environmental quality of shallow soil in different landforms and depths in the Nanling commodity grain base.

The average values of each index of soil samples at different depths in different geomorphic areas of the study area were calculated, and equal intercept transformation radar charts were drawn accordingly. The standard values of soil environmental quality given by GB15618-1995 were also drawn on the charts. In the drafting of the charts, instead of directly using each indicator's content, the data were multiplied properly to ensure that the contents of different indicators were on the same order of magnitude.

Then, these drawn radar charts were analyzed. First, a single indicator analysis was carried out to separately calculate the environmental quality levels of the soil indicators at different depths in different geomorphological areas. Second, the area of each equal intercept transformation radar chart, which represents the comprehensive soil environmental quality, was calculated (see Formula 1) and compared with that of the standard radar chart. Thus, the soil environmental quality of each evaluation unit was classified according to GB15618-2008.

1 $$S = \mathop \sum \limits_{1}^{n} A_{i} L\sin \frac{\theta }{2}$$

In the formula, *n* represents the number of indicators (*n* = 8 in this paper); *A*_*i*_ represents the value of the *i* indicator/axis; *L* represents “equal intercept” (*L* = 150 in this paper); $\theta$ represents the angle between two adjacent axes ($\theta$ = 45°, constant); $S$ represents the area of the radar chart.

## Data Availability

The datasets used or analyzed during the current study are available from the corresponding author on reasonable request.
